# Intervening alcohol marketing to reduce harmful alcohol use and lessons learned from the theory of changes: Case studies in Thailand

**DOI:** 10.1016/j.puhip.2021.100116

**Published:** 2021-03-31

**Authors:** Paibul Suriyawongpaisal, Sawitri Assanangkornchai, Udomsak Saengow, Ignacio J. Martinez Moyano, Roengrudee Patanavanich, Pongthep Wongwatcharapaiboon, Wichai Aekplakorn, Thanita Thongtan

**Affiliations:** a Department of Community Medicine, Faculty of Medicine, Ramathibodi Hospital, Mahidol University, Ratchathewi, Bangkok, Thailand; b Epidemiology Unit, Faculty of Medicine, Prince of Songkla University, Hat Yai, Songkhla, Thailand; c Center of Excellence in Health System and Medical Research, Walailak University, Tha Sala, Nakhon Si Thammarat, Thailand; d Argonne National Laboratory, Decision and Infrastructure Sciences Division, Argonne, IL, USA; e The University of Chicago, Consortium for Advanced Science and Engineering, Chicago, IL, USA; f Nan Hospital, Nan, Thailand; g Department of Internal Medicine, Texas Tech University Health Sciences Center, Lubbock, TX, USA

**Keywords:** Alcohol use, Theory of changes, Health policy

## Abstract

**Objectives:**

Globally, the burden of disease caused by alcohol use has been steadily increasing, including in Thailand. In this study, we aim to test the effectiveness of Anderson et al.‘s suggested three approaches to change the collective social norms, which comprise of: (1) providing information and an understanding about alcohol use behaviour, its causes and distribution; (2) focusing strategies on groups rather than individuals; and (3) strengthening supportive laws, regulations and approaches.

**Study design:**

We employed a mixed-methods approach. Evidence was gathered from literature review and in-depth interviews with key individuals who are responsible for community-based interventions to alcohol marketing strategies in Thailand.

**Methods:**

We chose to focus on two case studies in Nan and Surin provinces, where hospital-based longitudinal data (8 years) were available. Changes in casualties related to the harmful use of alcohol, resulting from interactions between community-based interventions and alcohol marketing during the time of annual festivals were investigated. We employed the theory of change (ToC) defined by Vogel to guide the data collection and analysis. We reviewed literature from online databases and grey literature to generate causal-loop diagrams.

**Results:**

We created a causal-loop diagram to describe the complexity of harmful alcohol use, its related factors, context, interventions and outcomes. Over the decade between 2006 and 2015, community-based strategies led to a substantial reduction of casualties (initially a 50% reduction, rising to an 80–90% reduction by the end of the study period) during the time of the festivals.

**Conclusions:**

The reduction in injuries and fatalities could be a result of the concerted actions, including legal sanctions of alcohol beverage sales and advertisement, and public education to raise awareness and impart knowledge of the harmful use of alcohol. The actions were organised by a coalition of civil society, health professionals, public authorities and community leaders using hospital-based data on the adverse effects of harmful alcohol use to mobilise political support at the provincial level. The availability of long-term financial support as a catalytic source of funds and the presence of a comprehensive alcohol control act enabled framing and mobilisation of local resources and political support.

## Highlights

• Theory of change and causal-loop diagram are practical tools for approaching the complex harmful alcohol use reduction.
• Intervening alcohol marketing needs a systematic approach in shifting the social norms.
• Community-based actions/campaigns associated with the decline of alcohol-related injuries in Thailand.

## 1 Introduction

The global burden of disease attributable to alcohol use has increased over recent decades, accounting for 3 million deaths and 132.6 million disability-adjusted life years (DALYs) in 2016 [1]. Thailand’s alcohol consumption per capita escalated from 0.26 ​L of pure alcohol in 1961 to 8.3 ​L in 2016,[1,2] paralleled with the growing alcoholic beverage market [3]. In 2016, the 12-month prevalence of alcohol use disorders in Thailand was 10% in males and 0.9% in females, the alcohol-attributable deaths from road traffic injuries were 6759 per 100,000 population and alcohol-related deaths from all causes ranked the highest in the Southeast Asian region [1].

Alcohol use was the second leading health risk cause of DALYs in Thailand, attributable for 12.4% of the total disease burden in 2014[4], which is 2.4 times greater than the global disease burden attributed to alcohol (5.1%) in the same year [5]. The commercialisation of alcohol has rapidly increased in recent decades [1] because the alcohol industry is very effective at marketing campaigns [6] and social media [7]. The influence of alcohol advertising on subsequent alcohol consumption has been observed in young people [8]. Transnational alcohol companies have expanded rapidly into low- and middle-income countries (LMICs), benefiting from weak government policy and market liberalisation [9].

In 2001, the Thai parliament adopted the Health Promotion Foundation Act to add a 2% surcharge to alcohol and tobacco excise tax [10]. Since then, sustained social movements have taken place through a well organised network of civil society, sponsored by the Thai Health Promotion Foundation (ThaiHealth), which is funded by the 2% surcharge tax. The social movements use the following five strategies: (1) enforcement of law to control alcohol use, such as alcohol advertising regulations; (2) countering branding of the alcohol industry by limiting and/or replacing alcohol sponsorships with a zero-alcohol advertisement in popular community events; (3) information systems to educate and raise awareness about alcohol use within society; (4) promoting alcohol-free zones, campaigns and networking to control alcohol consumption; and (5) building support systems to help individuals with alcoholism [11].

Following enforcement of the Alcoholic Beverage Control Act 2008 in Thailand to regulate direct alcohol marketing, alcohol companies created strategies through a legal loophole to advertise their products using the themes of goodwill, positive image, friendship, iconic lifestyle, and sponsoring sporting events or music festivals to attract adolescents and young adults [12]. Exposure to alcohol commercials is associated with the likelihood of starting alcohol drinking and increasing the amount of consumption [6]. Surveys conducted in 2017 showed that 10–30% of the Thai population were likely to drink alcohol if they were exposed to alcohol advertising images within the past 3 months [13]. To counteract alcohol marketing, Anderson et al. proposed three approaches to shift social norms in disfavour of harmful alcohol use, as follows: (1) providing information about the harmful use of alcohol; (2) focusing strategies on groups rather than individuals; and (3) strengthening supportive laws, regulations and approaches [6]. Likewise, Stop Drink Network, a network of civil society organisations, operates at both the local and national level. Hence, we believe that by investigating several case studies and the lessons learned from these, we can gain a greater understanding of how to change collective social norms to reduce harmful alcohol use. Thereby we chose to focus on how networks of local policy champions (mainly members of civil society) intervene in the alcohol industry’s marketing campaigns, shift social norms, and decrease the morbidity and mortality related to harmful alcohol use. In this regard, we employed a combined study method of a literature review and in-depth interviews of key individuals, focusing on locally relevant interventions and taking into account the three approaches suggested by Anderson et al. [14].

## 2 Methods

### 2.1 Settings

Local festivals have been successfully promoted year-round as an attraction to tourists across Thailand [15]. On the downside, it has been shown that increased alcohol consumption during the festivals is associated with increased alcohol-related traffic accidents [16]. Among several festivals that may be related to harmful alcohol use, a boat racing festival in Nan province and an elephant round-up event in Surin province were chosen as case studies relevant to the objectives of the current investigation.

The Nan boat racing festival has been held annually for over 200 years in the Nan province in Northern Thailand. September and October are the best time of year for the long-boat competition as the river tide is at its highest. The event attracts roughly 200 boats from 100 communities and about 20,000 tourists each year, generating approximately $US 700,000 in cash flow. The event is very popular as a result of the competitive sporting element and also the authenticity of this tradition [17]. The festival is part of a bigger picture of alcohol use in Nan province, which had a self-reported drinking prevalence of 42.4% in the region in 2004–2005 [18].

An elephant round-up event is held annually in November in Surin province, the Land of the Elephant, in Northeast Thailand. The elephant round-up begins with the marching of approximately 300 elephants through the streets, which is followed by an elephant breakfast and performances, including elephant talent shows, elephant capturing shows and elephant soccer. This event attracts roughly 700,000 tourists, generating $US 50 million in cash flow each year [19]. The festival is associated with widespread alcohol consumption and negative consequences related to its use, such as traffic accidents, violence and intoxication [20].

**Fig. 1:**
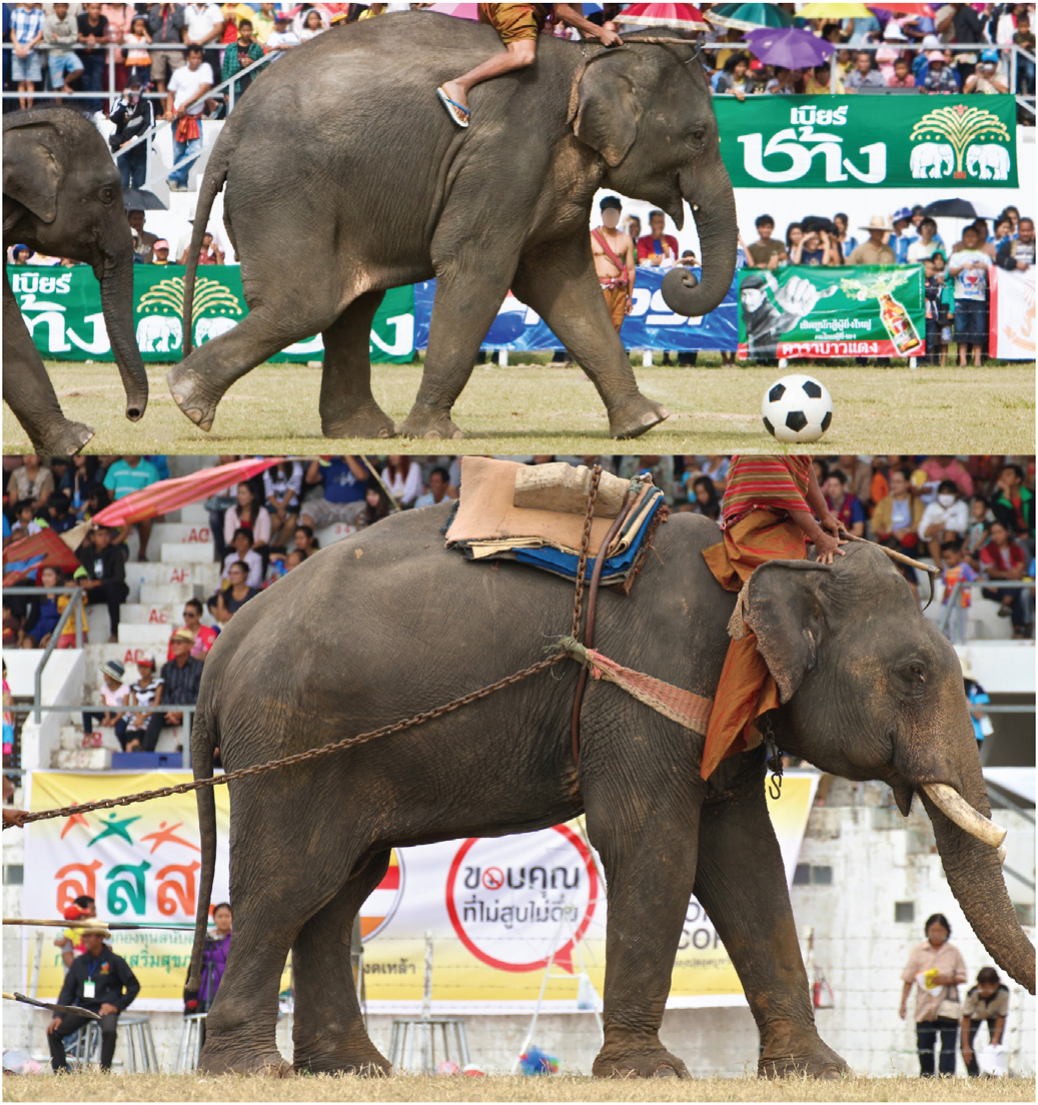
(upper) Alcohol sponsorship banner ads (green banners on the stand) at the Surin elephant round-up festival 2007; (lower) the alcohol sponsorship banner ads were replaced with no alcohol and smoking ads in 2008. (For interpretation of the references to colour in this figure legend, the reader is referred to the Web version of this article.)

Attempts have been made since 2008 to counteract harmful alcohol use during festival events in Surin and Nan provinces [21,22], which have been financially supported by ThaiHealth. Strategies have included implementing zero-alcohol campaigns, alcohol advertising bans, public education, alcohol cessation support, alcohol sponsorship replacement with a zero-alcohol advertisement (see Fig. 1) and responsible alcohol marketing regulations. These interventions are delivered by public agencies, community leaders and civil society organisations (Stop Drink Network) in a plan-do-check-act manner. Intervention design and execution were specific for the local context. For example, a stepwise approach was used in law enforcement to avoid jeopardising the long-term relationships between local authorities and local alcohol retailers, as well as to avoid resistance. The stepwise approach started with educating against unlawful practices before full-scale law enforcement actions were implemented. In terms of educating the general public, messages were blended through storytelling from alcohol-related victims and morbidity/mortality statistics were distributed throughout the community.

### 2.2 Data collection and analysis

We conducted a literature review, in-depth interviews with key individuals and thematic content analysis to identify issues, gather data, validate findings and expand our understanding of the complex nature of the designs and implementations of the campaigns against alcohol marketing over a decade-long period.

A review of the literature from online databases and grey literature was performed. Guided by the Theory of Change (ToC) [23] and Anderson’s approach [24], we searched the literature using Google, Google Scholar and PubMed with keywords such as alcohol, marketing, advertising, social norm, community action, survey, harmful use. Snowball searching of bibliographies was employed to supplement the first search strategy in terms of expanding our understanding of emerging issues and validating the findings from earlier search results. In the context of the present study, we considered alcohol-related casualties as a proxy outcome for harmful use of alcohol, which conceptually means a broad range of harmful effects in the short-term [25]. By making use of the proxy, we were aware of the limitation in demonstrating a relationship between the interventions and long-term consequences, such as chronic disease and chronic social impacts, from alcohol use [25].

Based on thematic content analysis of findings from the literature review, we conducted in-depth interviews with policy champions from Nan and Surin provinces, and campaign organisers (Table 1) to further expand our insight of the structure and processes of the designs and implementations of the campaigns. These individuals were purposively chosen according to their leading status stipulated in the Stop Drink Network website. This enabled us to realise and obtain the best available objective evidence on the outcomes of the campaigns from the policy champions. The evidence was extracted from hospital datasets regarding alcohol-related casualties seeking care during festival events.

**Table 1:** List of the key informants who were interviewed.

Major role and function	Type of Agencies	Number
Campaign organisers	Ministry of Health, NGOs, ThaiHealth	3
Policy champions	NGOs, Public hospitals	8
**Total**		**11**

NGO, non-governmental organisation.

**Fig. 2:**
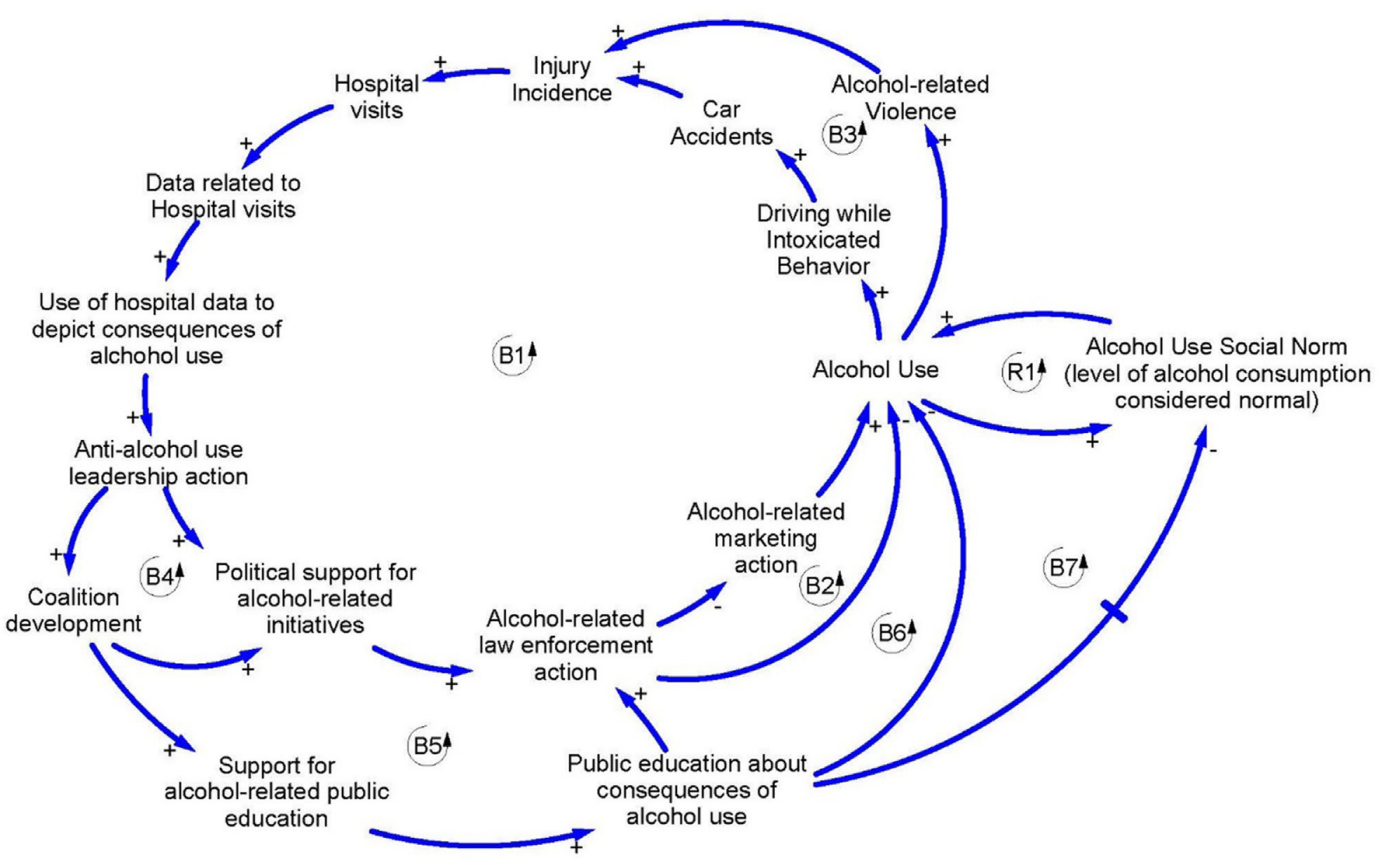
Causal-loop diagram using the theory of change (ToC) depicting harmful alcohol use and related factors. Arrows indicate the direction of implied causality between factors. Signs (‘+’ and ‘–‘) indicate the polarity of the relationship. A ‘+’ sign means that, all else equal, increases in the factor at the beginning of the arrow will result in increases in the factor at the end of the arrow. Similarly, a ‘–‘ sign means that, all else equal, increases in the factor at the beginning of the arrow will result in decreases in the factor at the end of the arrow. Reinforcing loop polarity (denoted by ‘R’ in the loop identifier) indicates the existence of a self-reinforcing (also called ‘positive’) feedback process. Balancing loop polarity (denoted by ‘B’ in the loop identifier) indicates the existence of a balancing (also called ‘self-controlling’ or ‘negative’) feedback process. Finally, the arrow with a crossed bar indicates delayed feedback [39,40].

We also made use of the in-depth interviews to finalise the causal-loop diagram. We used the ToC [23], Anderson’s approach [24] and causal-loop diagrams in backward mapping the feedback structure of harmful alcohol use reduction. The related factors, different stages of outcomes and interventions were analysed and discussed among the stakeholders using a group model building approach to get different points of view (see Fig. 2).

All the data were analysed using thematic content analysis by PS, WA and RP guided by the ToC. Disagreements were resolved through dialogue among authors. Finally, we presented our findings and causal-loop diagram to stakeholders and collected their feedback for final analysis and interpretation.

## 3 Results

### 3.1 Nan boat racing festival

Evidence from a 2013 cross-sectional study, using mixed methods design, surveying 500 contestants or sport team supporters’ focus-group sessions, and in-depth interviews involving 40 participants in 64 communities [26], revealed 86–95% of the contestants (n ​= ​410) or cheer-team members (n ​= ​90) reported drinking alcohol while driving home. These figures were higher than the highest prevalence of drink drivers (78%) at the provincial level in 2017 [27]. The figures were also in keeping with an earlier study [28] that reported on the direct observation of alcohol drinking among 7836 and 12,372 attendants in 2006 and 2007, respectively. Furthermore, the study documented the presence of an alcohol outlet every 3.2–3.5 ​m on the riverbanks.

However, recent evidence from a series of community-based surveys on the perceptions and opinions of stakeholders in Nan province indicates support of sustained desirable alcohol-reduction outcomes. In 2015, a survey of 477 stakeholders of the boat race festival (304 attendants, 57 organisers, 54 vendors and 62 boat race contestants) revealed a majority opinion in support of alcohol-free festivals in terms of preventing social violence, road accidents and crime, while enhancing attractiveness for tourists, recreation and the pursuit of local traditions [29]. Almost 60% of respondents considered alcohol industry sponsorship of the festival to be inappropriate.

### 3.2 Surin elephant round-up festival

In contrast to the Nan province case study, direct evidence of the behavioural outcomes and marketing activities in the Surin province are limited. A survey of 203 attendants in 2013 revealed that approximately 80% were supportive of law enforcement actions and campaigns against alcohol use [30].

Furthermore, evidence indicating positive responses to the interventions was found. Firstly, photographic evidence of alcohol sponsorship replacement at the Surin elephant round-up festival is presented in Fig. 1 (lower panel). Also, the same type of evidence was found for the Nan boat racing festival. On social media, a section promoting the ‘Nan Traditional Boat Race’ in the Bangkok Post, one of the current English newspapers of the country, included the message ‘alcoholic beverage restrictions’ [31]. Similarly, on the TripAdvisor (a large online travel platform) website for the UK, viewers were not presented with the words: alcohol, drink, beer, wine or beverage, on the page promoting the ‘Surin Elephant Round-up’ [32]. Also, local travel platforms, such as Thailandexhibition.com, do not carry any logos from the alcohol industry on the advertising page promoting the Surin festival [33].

Despite the apparent absence of evidence of alcohol sponsorship specific to Surin and Nan festivals, an official report from one of the biggest Thai alcohol industry companies specified an annual budget allocation of over 200 million Thai baht for athletic development and 1.3% of which was for Thai boat racing events in 2017 [34].

### 3.3 Causal-loop diagram

Fig. 2, using causal analysis embodied in a causal-loop diagram, depicts a network of interrelated factors describing interactions of the elements involved in the harmful use of alcohol. In the causal-loop diagram, eight feedback processes are identified (seven balancing and one reinforcing process). The main feedback process identified (‘B1’ in Fig. 2) shows the process of control of alcohol use via law enforcement action. As alcohol use increases, behaviours, such as driving while intoxicated or engaging in violence, increase leading to accidents, injuries and hospital visits. Hospital visits, in turn, are documented and produce reliable data related to the consequences of alcohol use, leading to a higher probability of action by anti-alcohol use leadership. This subsequently generates political support for alcohol-related initiatives and increases the level of law enforcement action related to alcohol use. Increased law enforcement action ultimately reduces alcohol use. This balancing process effectively controls alcohol use by direct intervention.

Additionally, increased law enforcement action, after an adjustment time, creates the conditions to reduce the marketing activity which subsequently results in a decreased level of alcohol consumption and a direct reduction of alcohol use (see ‘B2’ in Fig. 2). Such a process operates at a certain speed and with a certain strength, reducing alcohol use and its consequences. The B2 process, is necessarily slower than the B1 process because of the time it takes the marketing companies and campaigns to react to the increases in law-enforcement action and associated consequences.

Effective leadership not only yields political support for alcohol-related initiatives, but also, after a delay, increases the possibility of the development of coalitions among relevant stakeholders, which further increases political support for alcohol-related initiatives (see ‘B3’ in Fig. 2). Additionally, coalition development enhances support for public education related to the consequences of alcohol use. Public education about the consequences of alcohol use influences, after a time delay, law-enforcement action (see ‘B4’ in Fig. 2), alcohol use itself (see ‘B5’ in Fig. 2) and the development of a social norm around alcohol consumption (see ‘B6’ in Fig. 2).

As education around alcohol increases, the level of consumption that is considered ‘normal’ decreases in line with a greater understanding of the negative consequences of consumption. The level of alcohol use at any given point in time (short-term recognition of use) also influences the social norm (a long-term perception of what is ‘normal’) about alcohol consumption. As alcohol use increases, the social norm about consumption, after some time, also increases creating a dangerous reinforcing process that can act as a self-perpetuating trap (see ‘R1’ in Fig. 2). It should be noted that the term ‘normal’ is used to reflect the dynamic nature of the level of tolerance or acceptance for the level of alcohol use that the public has at any given point in time in relation to changes in the level of their understanding of the consequences of alcohol use.

If, through interventions and a greater understanding of the consequences of abuse in alcohol use, either the level of alcohol use or the social norm for alcohol use decreases, then the dangerous reinforcing cycle can become a virtuous cycle that, as time progresses, can lead to sustained reductions in alcohol use and a lower social norm reinforcing each other. In other words, by virtue of this reinforcing cycle, the desired outcomes related to preventing alcohol-related morbidity/mortality can be gradually met.

The interactions of the mechanisms, interventions and outcomes happened within the context of (1) the legal framework enabling the legitimacy of political decisions and support at the provincial level; (2) the availability of the hospital dataset enabling the contextualisation of locally-relevant knowledge; and (3) the availability of ThaiHealth’s financial support serving as catalytic funding for the intervention implementations and research funding for the generation of the indirect evidence in the longer term.

### 3.4 Festival casualties

**Fig. 3:**
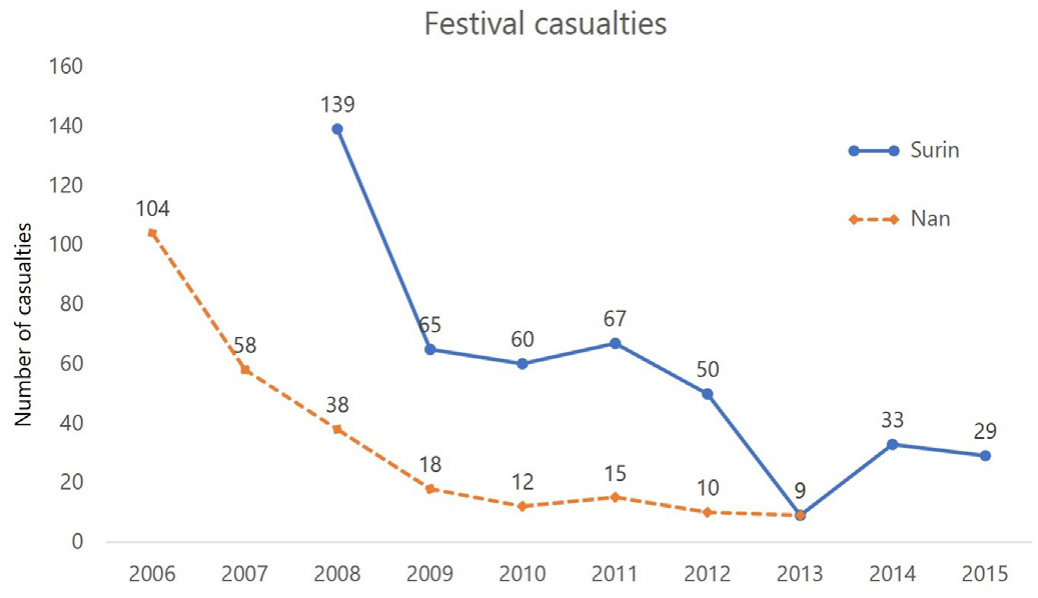
Trends in the number of casualties following interventions to alcohol marketing strategies at the Nan boat racing festival from 2006 to 2013 (dash line) and the Surin elephant round-up festival from 2008 to 2015 (solid line).

Using hospital statistics during the 2–3 days of the festivals each year, the number of hospitalised casualties was reported to be 104 for road traffic injuries during the Nan boat racing event in 2006 and 139 for all injuries during the Surin elephant round-up event in 2008. These data are prior to the Alcoholic Beverage Control Act 2008 when strategic interventions were implemented. The interventions have helped to bring the number of hospitalised casualties down steadily in the subsequent years, as shown in Fig. 3.

## 4 Discussion

After the strategic interventions in 2008, the number of festival casualties recorded in Nan and Surin hospitals has been steadily declining. In contrast, at a national level, injury surveillance has revealed stabilised trends of alcohol-related road traffic casualties visiting hospital emergency departments ranging from 60 to 44 per 100,000 population during the festival days of New Year or Song Kran (Thai New Year) from 2003 to 2010 [35].

To demonstrate how marketing within the alcohol industry might be effectively intervened in a middle-income country setting, our study used Anderson’s approach [24] to gather data guided by Vogel’s ToC [23] to explore the relationships between context, mechanisms, interventions and outcomes (see Fig. 2) by (1) providing information and an understanding about alcohol use behaviour, its causes and distribution; (2) focusing strategies on groups rather than individuals; and (3) strengthening supportive laws, regulations and approaches. This is a good example of how scientific knowledge can be contextualised or tailor-made to a specific context with several dynamic factors. Finally, making use of the causal analytical approach used here, embodied in the causal-loop diagram represented in Fig. 2, it is arguable that alcohol-related law enforcement action and public education are the major contributing factors to alcohol use reduction through several causal pathways. As means of intervention, education to the public about the consequences of alcohol use might require a longer timeframe to implement (due to several delayed feedback loops) than law enforcement actions. However, once public education about the consequences of alcohol use has had the opportunity to mature and influence the system, this intervention has the potential to more directly influence the social norm of consumption of alcohol use, which is critical to reducing actual long-term alcohol use (see ‘R1’ in Fig. 2).

In contrast to conventional academic approaches, such as experimental studies with randomised controlled trials or quasi-experimental studies beginning with the rigorous ideal design before intervention, case studies exemplify real-world situations where feasible trial-and-error occurs. The case studies described here provided concrete evidence for changing the collective social norms in preventing harmful use of alcohol (denormalisation).

However, concerning short- and medium-term outcomes, the inconsistency of the indirect evidence in the Nan and Surin cases raises a concern of validity and reliability of the results and raises some scepticism on the conclusion related to the effectiveness of the interventions. Using a difference-in-difference approach in analysing injury surveillance data, Udomsak et al. found repeated patterns of reduction in alcohol-related injury and death over a period of 6 years (2012–2017) as a result of alcohol abstinence campaigns in Thailand [36].

In 2015, a community-based survey of 2167 Thai Red Cross festival attendants in 15 provinces throughout the country, including Nan and Surin provinces, showed that 90.2% of attendants reported not drinking during the previous year’s festival and 86.2% reported the intention to not drink at the current festival [37]. These figures were greater than the proportion of visitors identifying themselves as social drinkers or regular drinkers (47.1%).

Comparing results from provinces with a zero-alcohol campaign (n ​= ​9) and without (n ​= ​6), the percentage of visitors reported witnessing alcohol drinking/selling were 15.8%/10.1% (p ​= ​0.037) and 12.4%/11.1% (p ​= ​0.519), respectively. Given that the strategies to counter alcohol marketing in these regions are similar to the approaches employed in our case studies and have been adopted by the same network of NGOs, the findings from this community-based survey suggest the possibility of upscaling the interventions to a national level. The increased use of the approaches employed in the Nan and Surin case studies may reflect the influence of the Thai Red Cross as a single umbrella organisation of the festivals, in addition to the NGOs’ influence.

### 4.1 Limitations

Like other studies relying on qualitative methods, our study raises concerns of validity and generalisability. We address this issue by using multiple sources of evidence to crosscheck findings and using feedback from stakeholders at the final stage of data analysis and interpretation. Although the implementation of the two case studies is not straightforward, findings from Udomsak et al. [36] and the survey of the Thai Red Cross festival attendants [37] provide support for upscaling the strategies on intervening alcohol marketing by correcting social norms in disfavour of alcohol use. Lack of specific evidence on short- and medium-term outcomes reflects an insufficient monitoring system from the beginning of the campaigns that made us rely on the existing grey literature. The decline of alcohol-related injuries in these two case studies might be due to two factors: (1) the increasing proportion of non-drinking visitors to the festivals; and/or (2) the effects of regression towards the mean. Nonetheless, considering the rather stabilised trends of the injuries during the New Year and Song Kran festivals over the same time periods, these explanations are quite unlikely.

## 5 Conclusions

Given the complexity of the causal system of interconnected psychological, behavioural, social, economic, legal and environmental factors leading to harmful alcohol use [38], it is practical to use a systems perspective to develop systems strategies to tackle the issue from multiple leverage points within the system. The community builder’s approach to ToC [23] organised by Anderson et al. is a method that policymakers can use to think about what is required to create desired social change. Given the broad range of harmful effects of alcohol use, focusing on acute harmful effects, such as alcohol-related injuries, could be a strategic choice to mobilise community actions as demonstrated in the present study. Finally, our study highlights the importance of careful monitoring and evaluation of strategies from the beginning as an integral part of the project.

## Ethical approval

The present study was approved by the Institutional Ethical Review Board of the Faculty of Medicine, Ramathibodi Hospital (ID 08-60-47), with the waiver of informed consent as the research involves no more than minimal risk to subjects.

## Funding

The authors acknowledge the 10.13039/501100009061Thai Health Promotion Foundation for financial support under contract No.60-00-1746, August 19^th^, 2017. Argonne National Laboratory’s work was supported by the U.S. Department of Energy under contract DE-AC02-065CH11357. The funders had no role in data collection, analysis, interpretation, writing of the manuscript or the decision to publish.

## Availability of data and materials

The datasets used and/or analysed in the current study are available from the corresponding author on reasonable request.

## Authors’ contributions

PS, RP, WA designed and conducted data collection and analysis as well as preparing the first draft of the manuscript. SA and US commented on the draft and provided additional literature to provide deeper and broader insightful narration. IJMM commented on the draft and provided the final causal-loop diagram based on the first draft from PS. PW commented on the draft and provided the original photos and figures. TT commented on the draft, worked on the comments from all authors and finalised the manuscript for submission. PS, SA, US, IJMM, RP, PW, WA and TT read and approved the final manuscript.

## Declaration of competing interest

The authors declare that they have no known competing financial interests or personal relationships that could have appeared to influence the work reported in this paper.
